# The path to impact of operational research on tuberculosis control policies and practices in Indonesia

**DOI:** 10.3402/gha.v9.29866

**Published:** 2016-02-25

**Authors:** Ari Probandari, Bagoes Widjanarko, Yodi Mahendradhata, Hary Sanjoto, Ancila Cerisha, Saverina Nungky, Pandu Riono, Sumanto Simon, Muhammad Noor Farid, Sardikin Giriputra, Artawan Eka Putra, Erlina Burhan, Chatarina U. Wahyuni, Dyah Mustikawati, Christina Widianingrum, Edine W. Tiemersma, Bachti Alisjahbana, on behalf of the Tuberculosis Operational Research Group (TORG)

**Affiliations:** 1 Department of Public Health, Faculty of Medicine, Sebelas Maret University, Surakarta, Indonesia; 2 Department of Health Promotion, Faculty of Public Health, Universitas Diponegoro, Semarang, Indonesia; 3 Centre for Health Policy and Management, Faculty of Medicine, Universitas Gadjah Mada, Yogyakarta, Indonesia; 4 Department of Biostatistics, Faculty of Public Health, Universitas Indonesia, Jakarta, Indonesia; 5 Department of Clinical Pathology, Faculty of Medicine, Universitas Atmajaya, Jakarta, Indonesia; 6 Department of Pulmonology and Respiratory Medicine, Faculty of Medicine, Universitas Indonesia, Jakarta, Indonesia; 7 Department of Public Health, Faculty of Medicine, Universitas Udayana, Denpasar, Indonesia; 8 Department of Epidemiology, Faculty of Public Health, Universitas Airlangga, Surabaya, Indonesia; 9 Sub-directorate of Tuberculosis, Ministry of Health, Jakarta, Indonesia; 10 KNCV Tuberculosis Foundation, The Hague, The Netherlands; 11 Department of Internal Medicine, Faculty of Medicine, Universitas Padjajaran, Bandung, Indonesia

**Keywords:** qualitative study, knowledge translation, research, operational research, tuberculosis, Indonesia

## Abstract

**Background:**

Operational research is currently one of the pillars of the global strategy to control tuberculosis. Indonesia initiated capacity building for operational research on tuberculosis over the last decade. Although publication of the research in peer-reviewed journals is an important indicator for measuring the success of this endeavor, the influence of operational research on policy and practices is considered even more important. However, little is known about the process by which operational research influences tuberculosis control policy and practices.

**Objective:**

We aimed to investigate the influence of operational research on tuberculosis control policy and practice in Indonesia between 2004 and 2014.

**Design:**

Using a qualitative study design, we conducted in-depth interviews of 50 researchers and 30 policy makers/program managers and performed document reviews. Transcripts of these interviews were evaluated while applying content analysis.

**Results:**

Operational research contributed to tuberculosis control policy and practice improvements, including development of new policies, introduction of new practices, and reinforcement of current program policies and practices. However, most of these developments had limited sustainability. The path from the dissemination of research results and recommendations to policy and practice changes was long and complex. The skills, interests, and political power of researchers and policy makers, as well as health system response, could influence the process.

**Conclusions:**

Operational research contributed to improving tuberculosis control policy and practices. A systematic approach to improve the sustainability of the impact of operational research should be explored.

## Introduction

*Operational research* (OR) can be defined as ‘the search for knowledge on interventions, strategies, or tools that can enhance the quality, effectiveness, or coverage of programs in which the research is being done’ (1). For the past 10 years, OR has been recognized as an important pillar of the control strategy for tuberculosis (TB) (2, 3).

Indonesia, a country with a high TB burden, has made efforts to promote the implementation of OR in the TB control program. The Tuberculosis Operational Research Group (TORG), formed by the National Tuberculosis Control Program (NTP) in collaboration with partners (e.g. KNCV Tuberculosis Foundation, the United States Agency for International Development [USAID], the Global Fund to fight AIDS, TB and Malaria), has been conducting OR capacity-building trainings in Indonesia since 2004. Details on the TORG's OR capacity-building activities and outputs have been published previously (4).

Dissemination of OR findings in peer-reviewed journals is only a single indicator of the success of OR; however, the ability of OR to contribute to improving policy and practices is even more important (5). This study aimed to explore how OR influenced TB control policies and practices in Indonesia between 2004 and 2014.

## Materials and methods

### Research settings

Indonesia is a middle-income archipelago in Southeast Asia consisting of 34 provinces. Since 2001, the government has applied a decentralized policy system. For TB control, this decentralization implies the sharing of responsibilities among the central, provincial, and district governments. The district government conducts basic management of TB control, including distribution of anti-TB drugs and laboratory reagents, training, supervision, monitoring, and evaluation of health facilities. The provincial government trains, supervises, monitors, and evaluates the districts. The central government is responsible for the development and supervision of standards related to TB, as well as the provision of quality TB drugs and laboratory consumables. The TB control program is integrated into the health care system, including primary health centers, lung clinics, and hospitals (2).

Since medical schools and/or public health schools commonly exist in the capital of each province, the TORG selected provincial OR groups for capacity-building purposes. Each OR group consisted of two to three researchers from academic institutions (‘academic-based researchers’) and one to two TB program staff (‘program-based researchers’). The process of capacity building has been described previously (4).

### Research design

This was a qualitative study conducted between March 2014 and December 2014. Data were collected by thorough in-depth interviews and document reviews.

### Informants and sampling

**Fig. 1:**
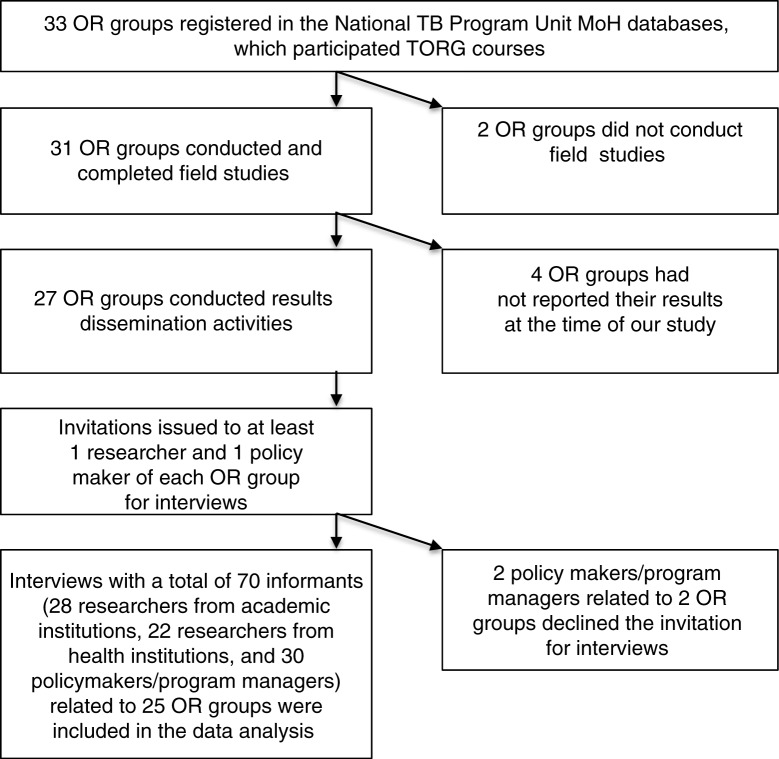
Selection of operational research projects and informants in this study. OR: operational research; MoH: Ministry of Health; TB: tuberculosis; TORG: Tuberculosis Operational Research Group.

By February 2014, 33 provincial OR groups had participated in the TORG proposal development workshop. The newest province, which was established in late 2012, was not involved in the TORG when this study was conducted. Only 31 groups conducted a field study. Two OR groups experienced teamwork problems and did not continue with the field study. Four OR groups were still in the data analysis phase and had not completed the project. Therefore, we included 27 OR groups in this study (Fig. 1).

We obtained a list of researchers who had participated in any of the TORG proposal development workshops held between 2004 and 2014 from the NTP. We selected the principal researcher and/or another co-researcher from each OR group. Relevant policy makers or program managers (i.e. hospital managers, heads of Communicable Disease Control (CDC) at the provincial health offices, district level TB program staff, and NTP focal points) were selected by snowball sampling based on information from the researchers.

### Data collection and analysis

We developed the guidelines of in-depth interviews according to the framework of Hanney et al. (6). The interview guidelines extracted information regarding the following:

- Results and recommendations of the OR
- Follow-up actions after results were disseminated
- Influence of OR upon policy and/or practice changes, as well as means of support and impediments

We collected OR project reports and harvested information on the recommendations from the study projects. This information was used for triangulation of data from interviews to assess the implementation of the OR project recommendations.

We excluded the OR projects of two provinces from the analysis since we were unable to obtain information from the policy makers’ perspective. Ultimately, we included interview data from 50 researchers and 30 policy makers in the analysis. The researchers consisted of 28 academics, 17 provincial health office staff members, 2 district health office staff members, and 3 hospital staff members. Policy makers included 4 national TB programmers, 21 provincial CDC/TB programmers, 3 district CDC/TB programmers, and 2 TB health service coordinators.

### Trustworthiness

To improve accuracy (7), this study applied triangulation of data sources (interviews and document reviews) and peer debriefing strategies to TORG members. AP and HS performed most of the interviews, whereas SN and AC were responsible for writing verbatim transcripts within 48 h. AP and SN checked the accuracy of verbatim transcripts before analysis. The preliminary results of the analysis were discussed among all the authors.

### Research ethics

This study received ethical clearance from the Committee of Research Ethics of the Faculty of Medicine of Universitas Sebelas Maret, Indonesia. The interviewers provided information about the study via emails and phone calls before conducting the interviews, and the informants provided verbal informed consent before the interviews. The interviews were taped, and informed consent was recorded. Transcripts of the interviews were anonymized, as were analyses and data interpretation/presentation.

### Data analysis

Content analysis (8, 9) was used for data interpretation. The authors read the verbatim transcripts several times in order to explore the response codes, which contained manifest meanings (i.e. obvious content, or meanings that are self-evident) or latent meaning (i.e. the indirect underlying message of the text). Codes sharing a commonality were grouped into a single category. A theme represented the link between categories. We used Open Code 4.02 software (10) to facilitate analyses, the results of which are illustrated in Tables 1 and 2.

**Table 1:** Example of data analysis

Topic	Meaning unit	Code	Sub-category	Category	Theme
How the findings of the study results were disseminated	We disseminated the results by inviting all heads of district health offices in Banda Aceh Province.	Result dissemination	The milestones	The path	The path to impact
Follow-up actions after results of OR were disseminated	There was an order [to implement the OR project recommendation] from the provincial health office, i.e. a letter circulated to private clinics and hospitals.	Policy development			
	We presented the OR results and recommendations in meetings, both national and district level.	Advocacy meetings			
	We were invited to the provincial Monev [monitoring evaluation] meeting in 2010 to discuss strategies of accommodating the OR results that were disseminated in 2008. The draft [of the policy document] was completed in 2011.	Time of policy document development	Time		
	I think it [the implementation of the OR recommendation] took approximately 1 year in the study district.	One year of implementation; unsustainable action	Sustainability of impact	The impact	
Influence of the ORs on TB service, program practice, and/or policy	We have ideas on what we should do. The OR results strengthened them [the ideas of the policy makers].	Strengthening current policy/practice	Spectrum of impact		
Factors perceived as important to the process of knowledge translation of the ORs	There was a change in leadership at the health office [in the district]. I foresee that innovations [from the OR results] will not be sustained.	Changes of policy makers	Policy makers	The keys	
	I am a bit frustrated … Yes, really frustrated. It [the OR recommendations] should be supported. I am just a staff member, I can only grumble.	Power of program-based researcher	Researchers		
	We could do these … because we have national and provincial policy support.	Support of policy	Health systems		

OR: operational research; TB: tuberculosis.

**Table 2:** Summary of analysis: codes, sub-categories, categories, and theme

Theme	The path to impact						
	
Category	The path		The impact		The keys		
	
Sub-category	Milestones	Time	Spectrum of impact	Sustainability of impact	Researchers	Policy makers	Health system response
Code	Preliminary advocacy; dissemination seminar Policy-brief documents Preliminary policy document development Letter of commitment Letter of recommendation Follow-up Formal advocacy meetings Citation of OR results Enhanced policy document Hospital regulation Memorandum of understanding	Time of policy document development Time of advocacy	Domain of impact Innovation New policy/practice Evidence-informed policy Improvement of existing policy/practice Scope of impact	Unsustained actions One year of implementation Several months of implementation Four years of implementation Replication to other districts	Collaboration of OR group Cohesiveness of OR group Communication skills of researcher Intensive contacts with policy maker Confidence of researcher to approach Confidence of researcher to persuade Power/authority of program-based researcher Change of job position of program-based researcher Researcher's skills in preparing contingency Researcher's advocacy skills	Lack of confidence in policy makers’ ability to deploy innovation Enthusiasm of policy makers Appreciation of policy maker Political power of targeted policy maker Perceived priority of policy maker Perceived feasibility of OR recommendations by policy makers Changes of policy maker	Budget Financial support Logistic matter Support of policy and regulation Relevant guideline Changes of program field staff

## Results

The 25 OR projects included in the analysis varied in topics and study designs (Table 3). Twenty-two OR projects were oriented to the improvement of TB program implementation at the provincial, district, or health service center level, while three OR projects targeted national level TB program policy improvement.

**Table 3:** Characteristics of operational research projects included in the present analysis

No	Title of study (year of study)	Study design	Province	Recommendations*	Implementation of recommendations**
Topic: Community-based TB case findings					
1.	The effect of health promotion to informal community groups in detecting TB suspects at Gowa District (2007)	An experimental study	South Sulawesi	To involve informal community champions to find presumptive TB cases and to refer to primary health centers.	Provision of financial incentives for health community cadres for every TB case that was identified by the cadres. One year continuing implementation of the intervention in the study district.
2.	The involvement of the Acehnese local community champion in TB control (2009)	An experimental study	Aceh	To involve the Acehnese community champions in the education of the community on TB and presumptive TB case identification.	A pilot project of the involvement of the community champions in TB education and TB presumptive case identification in a district for 1 year.
3.	The role of religious leaders in increasing pulmonary TB case notification (2010)	An experimental study	East Nusa Tenggara	Training of religious leaders in educating the community about TB. Provision of TB information materials.	Provision of TB educational materials.
4.	Involving the traditional market community in identification of suspected TB cases (2010)	An experimental study	Southeast Sulawesi	The provincial health office should involve the traditional market community in identification of suspected TB cases.	None.
Topic: Treatment-seeking behavior of people with symptoms of TB					
5.	Treatment-seeking behavior of TB patients (2005)	A qualitative study	Yogyakarta	The national TB control program should collaborate with hospitals and private medical practitioners. TB education in the community to increase access to DOTS health facility. Active TB case findings within community.	Stepwise training for hospitals on standardized TB case management including recording/reporting the cases to the district health office. The involvement of private medical practitioners in TB control in a district. Provision of TB education materials by the health promotion division of the Provincial Health Office. Training for primary health care staff on TB case detection. The implementation of active TB case discovery in the city of Yogyakarta.
6.	Treatment-seeking behavior of community and TB patients (2007)	A cross-sectional study	Lampung	Training of midwives and nurses in presumptive TB case identification.	Training of cadres on presumptive TB case identification in a sub-district (2007).
Topics: TB services at primary health care centers					
7.	TB control program performance in primary health centers (2007)	A cross-sectional study	North Sumatera	Improvement of lab facilities, including the lab waste system.	None.
8.	Treatment compliance of TB patients treated under the DOTS strategy in primary health centers (2008)	A case–control study	Papua	Education for patients and drug administration observers. Development of IEC materials in the Papuan language.	The production of IEC materials/media in the Papuan language (2011). The use of the materials for education of TB/HIV patients (2011).
9.	The effectiveness of contact tracing in increasing TB suspect identification (2009–2010)	An experimental study	Bengkulu	To conduct contact tracing, as an additional strategy to passive case discovery of TB. A cost-effectiveness evaluation of contact tracing strategy. Development of district level policy to support contact tracing.	Implementation of contact tracing as an additional strategy to passive case discovery of TB (2010). A letter was circulated from the provincial health office to district health offices in Bengkulu Province and primary health centers regarding the recommendation to conduct contact tracing (2010).
10.	TB suspect identification and TB case notification by enhancing the satellite public health center network on Haruku Island (2010)	An experimental study	Central Maluku	The development of networks between satellite health centers and main health centers. Provision of logistics.	Training of trainers on the development of networks between satellite health centers and main health centers (2014).
11.	Knowledge, attitude, and commitment of primary health center staff on TB program (2011)	A cross-sectional study	East Kalimantan	Dissemination of the DOTS strategy to hospitals. Hospitals should prepare for human resources responsible for the implementation of DOTS strategy at hospitals. The development of SOPs for DOTS strategy implementation in hospitals. Routine coordination.	Benchmarking visit to provincial health office and a hospital of West Sumatra, on the implementation of DOTS strategy.
Topic: Public–private mix for TB control					
12.	Implementation of DOTS strategy in hospitals (2005)	A mixed methods study	Central Java	To develop collaboration between specialist and other medical staff at hospital.	Guidelines and SOPs of DOTS strategy implementation in hospital.
13.	Implementation of directly observed treatment short-course strategy in hospitals (2007)	A qualitative study	South Kalimantan	The hospital should appoint specific (senior) TB staff to monitor the implementation of the DOTS strategy. Training in the DOTS strategy.	None.
14.	The contribution of private medical practitioners to presumptive TB case identification and referral (2007–2008)	A case–control study	Bali	Private practitioners should be involved in TB control, mainly referral of TB suspects to primary health centers.	A pilot study examining the involvement of private medical practices and non-governmental organizations in TB control (in 2009). A simplified TB recording and reporting system for private medical practices. A reward system from the Indonesian Medical Association for private medical practices who refer TB patients to primary health centers.
15.	Readiness of DOTS strategy implementation at hospitals (2009)	A qualitative study	Banten	To increase the commitment of hospitals in the implementation of the DOTS strategy, e.g. through the production of decision letters, job descriptions, and SOP and infrastructure support. Routine supervision by district health office. Coordination with district health office to intensify referral of cases from hospitals to primary health centers.	Production of letters of decision to establish a DOTS strategy implementation team at one of three hospitals in the study.
16.	The implementation of the ISTC in hospitals (2009)	A cross-sectional study	DKI Jakarta	To prepare human resources, supplies, and instruments for sputum tests at hospitals. To improve joint supervision between the provincial health office, hospital association, and professional organizations. To develop a memorandum of understanding between the provincial health office, hospital association, and professional organizations on ISTC implementation. Dissemination of the DOTS strategy among staff in hospitals. Inclusion of DOTS strategy implementation in national hospital accreditation.	The provincial health office conducted trainings on the DOTS strategy in hospitals (2010). Dissemination of the DOTS strategy by writing a letter to all private hospitals in the province (2010). Dissemination of the DOTS strategy in hospitals during three monthly monitoring evaluation meetings for all district TB program staff. The inclusion of the DOTS strategy in the national hospital accreditation (2012).
17.	Association between treatment observer characteristics and defaulting from TB treatment in Hasan Sadikin Hospital, Bandung (2009)	A case–control study	West Java	The presence of TB drug observers during patients’ visits to the hospital so that the provider can educate TB drug observers. Improvement of the pathway of TB services in hospitals by educating TB drug observers.	Education to TB patients and drug observers approximately once a week during the busiest day at the outpatient unit at Hasan Sadikin hospital. The education was conducted by residents of the pulmonology department. The Department of Pulmonology initiated the education without any changes to hospital SOPs regarding TB services.
18.	Development of a network between private laboratory and private medical practices in implementing the DOTS strategy (2010)	A qualitative study	West Sumatra	Diagnostic tests for TB in private labs should use sputum tests. The dissemination of information on proper suspect criteria, sputum specimen collection, and sputum tests. Private labs should report TB cases every month or every 3 months.	Training in private labs with province budget. A network of private labs and a system of reporting from private labs to Dinas Kesehatan was established and implemented.
19.	The effectiveness of TB education through SMS on treatment compliance among TB patients in hospitals (2011)	An experimental study	Central Java	The use of SMS technology to provide education to TB patients during treatment.	The adoption of an SMS-based educational system by TB patients in a district.
Topic: TB lab quality					
20.	Low quality of sputum specimens for TB diagnosis and its factors (2005)	A cross-sectional survey	Central Java	Training for providers on TB sputum specimen collection. Education for patients on how to collect sputum.	Trainings for laboratory staff (2006–2014)
21.	Quality of sputum tests in public health centers (2010)	A cross-sectional study	Jambi	Collaboration between TB program staff and laboratory staff at primary health centers. Monitoring of the recommended collaborative work by heads of primary health centers. Improvement of lab infrastructure. Supportive supervision by district health office. Assistance of provincial health office to district TB staff in managerial skills. Advocacy to head of district health office in Jambi Province.	Continuation of the collaborative work between TB program staff and laboratory staff at primary health centers for some months in 2011.
22.	The effectiveness of training on TB microscopy diagnosis among laboratory staff (2011)	An experimental study	West Nusa Tenggara	Continue to conduct training of the laboratory staff at the primary health centers.	Training for lab staff at primary health centers. Logistics and equipment provision. Laboratory staff workload reduction.
Topic: TB in children, MDR-TB, TB-HIV					
23.	Delay in treatment among MDR-TB patients (2011–2012)	A cross-sectional study	DKI Jakarta	Decentralized treatment from hospital to primary health care level. Incentives for MDR-TB patients to support the negative impact of loss of income during the treatment.	The MDR-TB program plan to decentralize MDR-TB treatment at primary health care centers (2013).
24.	Assessment of TB pediatric scoring chart (2011–2012)	An experimental study	DKI Jakarta	Training and technical assistance to general practitioners at primary health centers on a pediatric TB diagnostic scoring system.	Training of medical doctors in primary health centers on pediatric-TB-scoring chart (2013). Inclusion of pediatric TB cases on the agenda of routine monitoring and evaluation meetings (2013–2014). Continuing technical assistance on the use of scoring charts for pediatric TB diagnosis (2014).
25.	The improvement of TB-HIV collaboration in Hasan Sadikin Hospital, Bandung (2011)	Action research	West Java	Training for hospital staff on TB-HIV collaboration. Supervision of TB-HIV collaboration. Improving the reporting system.	The sub-directorate TB MoH planned and implemented the following: (1) Supervision and technical assistance to provinces with under-achievement of TB-HIV program target indicators (2) Workshops on recording, reporting, and strengthening anti-TB-HIV collaboration efforts (3) Routine meetings of the national TB-HIV team These three activities were not perceived to directly impact the operational research. Intensified communication between the TB and HIV units at the hospital (2013–2014). A modified model of integrated TB-HIV services at the hospital (2013–2014). A TB-HIV team at the hospital level, which is responsible for the management of services for TB-HIV patients (2014). Improved reporting system of the TB and HIV units (2013–2014). Improved indicators of the TB-HIV collaboration program (2013–2014).

* Information was collected from the OR study reports

** Information was collected from the interviews with policy makers/program managers and researchers. DOTS: directly observed treatment short-course; HIV: human immunodeficiency virus; IEC: information, education, and counseling; ISTC: International Standard for Tuberculosis Care; MDR-TB: multidrug-resistant TB; MoH: Ministry of Health; SMS: short message service; SOP: standard operating procedure; TB: tuberculosis.

The analysis revealed a ‘path to impact’ theme that consisted of three categories: the impact, path, and keys. The impact described issues pertaining to the contribution of OR projects to relevant TB policies and practices. The path revealed the processes from the end of the OR field study to the success/failure of OR projects in contributing to TB policies and practices. The keys represented relevant factors that supported or hindered the implementation of the OR projects’ recommendations.

### The impact

#### Spectrum of impact

Descriptions of OR impact are presented in Table 3. Our analysis found that most OR projects (22 of 25) contributed to TB policy and practices in a spectrum of domains and scopes (Table 4). The spectrum showed that OR project contributions to TB policy and practices could be divided into two domains: 1) the impact of the OR project on the development of new TB program policies or practices and 2) actual evidence that the OR project improved or reinforced existing policies or practices. The impact of the OR project was observed on the national, provincial, district, and health facility center levels.

**Table 4:** Spectrum of OR impact in TB control program policies or practices

		Domain	
		
		Strengthening current TB control program policies or practices	New TB control program policies or practices
Scopes	National	Decentralized treatment of MDR-TB at primary health services Inclusion of TB service in the hospital accreditation assessment	NA
	Province	Intensified trainings on pediatric TB diagnosis scoring system The implementation of pediatric-TB-scoring charts at TB services of primary health centers Validated MDR-TB data Intensified trainings on laboratory Intensified trainings on DOTS strategy to hospital staff	Reward system for private practitioners’ contributions to TB control Modified reporting form of TB suspect identification by private practitioners Short message service–based TB education
	District	Intensified trainings on laboratory Intensified trainings on DOTS strategy to hospital staff Production of TB education materials	TB education materials in local language Innovative approaches to TB case finding
	Health facility	A modified model of integrated TB-HIV services	Collaborative work between the TB program staff and lab staff at primary health centers Standard operating procedure on collaborative TB-HIV services

NA: not applicable.

Thirteen OR projects related to the first domain of impact. For example, the OR project in Bali Province led to the development of reward systems for private practitioners’ contributions to TB suspect referrals. Additionally, the OR project on TB–HIV collaboration at a teaching hospital in West Java Province triggered the development of standard operating procedures for TB–HIV collaboration at the hospital level.

Nine OR projects involved strengthening current TB control policies and practices upon evaluating the strengths and weaknesses of the activities of TB control programs. Some OR projects produced recommendations that the TB control program had already implemented, although the studies were not yet completed. For instance, the OR project on pediatric TB was initiated concurrently with the development of the national pediatric TB guideline. However, the guideline was finalized while the OR project was still ongoing. The OR duration and the dynamics of the TB control program activities were important factors, as expressed in the following quotation:

The multidrug-resistant TB program was very dynamic. Because our OR project had a long duration, the recommendations from our OR could not keep up with the continuously shifting dynamics of the program. (Academic-based researcher, Jakarta Province)

#### Sustainability of impact

The recommendations of most OR projects included in our study resulted in changes to policies or practices that lasted for various lengths of time. For example, the recommendation from the OR project in Jambi Province that the work of the TB program staff and laboratory staff be integrated at the primary care level was only maintained for less than a year after the results dissemination seminar. The recommendation of involving community members in active TB case detection in Aceh Province was maintained for approximately 1 year. The recommendation to award accreditation points to private practitioners involved in TB case detection in Bali Province was maintained for a longer period. OR recommendations were less sustainable when they concerned innovations of policies or practices instead of recommendations to strengthen existing policies and practices.

### The path

#### The milestones

**Fig. 2:**
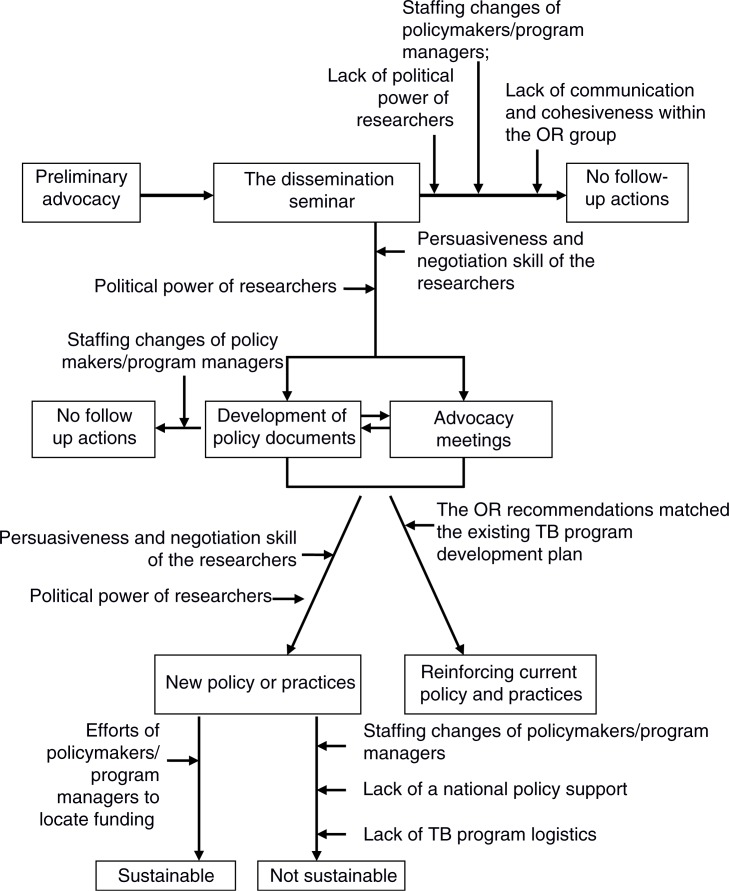
The process from operational research dissemination to impact, including support mechanisms and impediments. OR: operational research; TB: tuberculosis.

The overall process of translating knowledge obtained through OR into influence on TB program policy and practice is presented in Fig. 2. Milestones include the following: 1) preliminary advocacy; 2) the dissemination seminar; 3) development of policy documents; 4) advocacy meetings; and 5) new policies and practices, or reinforcement of current ones.

Some OR groups informally shared preliminary findings before the official dissemination seminar to raise interest among relevant policy makers. Some OR groups also created policy-brief documents.

Approximately 1 year after commencing field work, all OR groups conducted an official dissemination seminar for stakeholders from multiple disciplines who may have had the capacity to implement, or catalyze implementation of, the study recommendations. There were three types of policy makers invited: health facility level program managers (i.e. heads of hospitals, heads of lung clinics, and TB service providers); provincial or district level managers (i.e. heads of health offices, heads of CDC units, TB program staff, provincial/district health planning and budgeting bureau staff, and members of the provincial house of representatives); and national level managers (i.e. national TB program staff). During the dissemination seminars, the OR group typically presented the results and recommendations of the study; discussions with stakeholders then followed.

In general, the policy makers accepted the OR results and recommendations, since these were regarded as relevant, practical, and having the potential to improve TB program performance.

I think the results [of OR] are useful. Other [district] TB program coordinators could accept them [the results and recommendations]. Yes, these [the recommendations] are feasible … (TB program officer, Bengkulu Province)

It was common for policy makers to develop preliminary policy documents (e.g. a letter of commitment to support the OR recommendations) as a follow-up to the dissemination seminar. However, the preliminary policy documents did not guarantee that the policy makers would deploy the recommendations of the OR projects (Fig. 2). Only a few OR projects led to the development of enhanced policy documents, such as a signed memorandum of understanding between institutions or a change in hospital regulations. Usually, development of the enhanced policy documents was preceded by a series of advocacy meetings.

For some projects, researchers or/and policy makers conducted additional advocacy activities to obtain supporting policy papers and funding, thereby ensuring the application of the OR recommendations. In general, the policy makers/program managers requested that the researchers conduct additional presentations about the OR results and recommendations in a formal meeting (such as the provincial TB surveillance meeting). Otherwise, the policy makers/program managers promoted the recommendations of the OR projects to all relevant facilities under their authority. Some researchers highlighted evidence from the OR project in their presentations at various forums.

#### Time

The time between the dissemination seminars and the deployment of the recommendations ranged from months to years (Table 3). Complex actions related to policy (e.g. reward systems and national accreditation) took longer than those related to practices (e.g. training, modified recording/reporting forms, and education of TB patients).

### The keys

Researchers, policy makers/program managers, and health system personnel form the keys to translating knowledge from OR projects into changes to TB program policy or practices. The roles of these individuals in OR contributions and achievement of milestones in TB policy or practices are presented in Fig. 2.

#### The researchers

Good collaboration and cohesion existed in the OR groups that impacted TB program policy or practices. In contrast, a group member leaving for a new job could impede follow-up on the OR recommendations (Fig. 2). One academic-based researcher mentioned, ‘We could not follow up [on the recommendations] intensively because of the move of the program-based researcher to another position’ (Banten Province).

OR projects that led to program or policy changes tended to have program-based researchers with sufficient power and confidence to approach and persuade the policy makers and/or program managers. Conversely, program-based researchers who had no relevant authority felt less capable of persuading TB program policy makers.

Communication and advocacy skills, as well as contingency planning, were also key to the successful influence of an OR project on policy and practice (Fig. 2) as exemplified in the following quotation:

We coordinated and tried to work with the local Indonesian Medical Association. We had conducted activities [to involve private medical practitioners] together [with the Indonesian Medical Association]. However, the results did not satisfy our expectations, as most private practitioners did not attend the meeting. Therefore, we changed our strategy the following year. (Program-based researcher, Bali Province)

#### The policy makers

A combination of enthusiasm and political power on the part of the policy makers was shown to be an important facilitator of achieving impact (Fig. 2). Those policy makers/program managers who took a personal interest in the OR findings and recommendations encouraged their peers to pursue the implementation of the recommendations. Such advocacy was successful if the policy maker/program manager had sufficient power to push for the deployment of the OR project recommendations.

Changes of high level policy makers/program managers (e.g. head of district or head of provincial/district health office) or middle level policy makers/program managers (e.g. TB program staff at provincial or district levels, or heads of primary health centers) were often mentioned as obstacles to implementing recommendations for policy or practice changes. After a change of policy maker, it was more difficult for the researchers to ensure that the OR recommendations were implemented. Hence, such changes were also barriers to the sustainability of the impact of the OR.

#### Health system response

Availability of a relevant national policy document or guideline was another key factor regarding implementation of innovative recommendations from the OR projects (Fig. 2). Some of these recommendations were not supported by existing regulations. For instance, the recommendation to apply enhanced case detection involving community members (such as religious leaders, women, or the traditional market community) could not be implemented in the TB control programs of some provinces due to the lack of a relevant national policy on an active case detection approach, which is required to formally promote such strategy. At the time that these recommendations were launched, the NTP still prioritized the passive case detection approach; the district/province level policy makers were reluctant to deploy innovations that were not supported by any existing higher level policy:

When we disseminated our study results to other districts, they [the district TB program staff] were questioning how they could implement the recommendations [of community-based active case finding] … Until now, the national TB control guideline [has] only [included] the passive case detection approach … (Program-based researcher, Aceh Province)

Availability of financial support was another key to facilitating the impact of OR projects (Fig. 2). Some study recommendations could not be deployed due to the lack of funding. However, a few policy makers were able to resolve financial barriers. In such cases, the enthusiasm of policy makers for the OR results motivated them to locate alternative sources of funding.

The preparedness of the Ministry of Health to respond to logistical provisions related to enabling application of OR project recommendations was another challenge. For example, the recommendation to implement active TB case detection by so-called community champions in Aceh was followed, and the numbers of TB cases reported by primary health centers increased. This consequently required additional drugs and laboratory supplies, something the logistics planners had not anticipated.

Finally, staffing changes hampered the sustainability of the OR-recommended actions that involved TB program field staff. For example, the impact of the integrative work of the staff from the TB program and the laboratory in Jambi Province was diluted mostly due to the turnover of the TB program staff at the primary health centers.

## Discussion

Our study provides empirical evidence regarding the impact of OR projects on changes of policies and practices; similar impacts have been described previously for other areas of focus (11, 12). The OR projects produced a range of influences on TB programs (Table 4), which was expected given the wide spectrum of possible OR projects (13). Our findings showed that the OR projects contributed chiefly to the improvement of TB program practices at the provincial and district levels. OR can vary because of the heterogeneous settings of Indonesian provinces. Therefore, the TORG implemented a local context approach when devising the research questions (4), especially as OR is a form of research investigating problems in the health program up close. Hence, OR potentially avoids the problem of unnecessary wasteful research (14).

The mechanisms of the influences of OR projects on TB control policies and practices are complex and nonlinear (Fig. 2), as has been argued by others (15, 16). Our study showed that the TORG's approach of combining academics and health staff into a single OR group helped the OR projects to achieve greater impact. As stated by Grimshaw et al. (17), the inclusion of health staff as program-based researchers has helped the OR projects to contextualize the research problems, increase the applicability of the recommendations, and facilitate the communication of research findings to the relevant policy makers and program managers. However, careful selection of the health staff is important, as those with excellent communication and advocacy skills as well as sufficient influence are critical assets. Our study shows that the time elapsed between the OR projects' duration and TB program development is a challenge. In particular, shorter study durations are needed for the development of new policies and practices based on innovative ideas emanating from OR projects. In our study, some policy makers or program managers perceived that the OR recommendations only came after the introduction of new policies or practices. Although this was true for a number of the studies included here, such studies still yielded evidence useful to policy makers and program managers regarding whether their policy changes were in fact improving the program. Prompt communication of the OR results to policy makers and managers is essential (12). Khotari et al. (18) also suggested that interaction between producers (i.e. researchers) and users (i.e. program managers) increases the users’ understanding of the research and enhances the likelihood that the research findings will be valued.

The recommendations from some OR projects in our study could not be maintained. This was sometimes due to the irrelevance of the recommendations to existing policies; at the time study results were disseminated, there would already be a new and better policy. Therefore, process dynamics and policy relevance should be taken into account when the researchers plan for OR; as Ioannidis argued, fine-tuning between research and existing policy should be considered (19).

Our study found that the implementation of OR project recommendations may require a broader health system response, such as the availability of overarching policies and additional resources. Others have also shown that financial and human resource constraints impede the use of research evidence in decision-making (20). Again, prompt communication between researchers and policy makers/program managers should be encouraged to improve the preparedness of the health system to implement OR project recommendations (6).

Our study evaluated OR projects facilitated by the TORG in the context of decentralized health systems; this limits the generalization of our findings. Nevertheless, we believe that our study provides evidence for the impact of the OR projects on policies and practices. Moreover, the lessons shared in this paper could be of use to ongoing initiatives involving OR capacity building in other countries.

## Conclusions and recommendations

Our study concluded that OR contributed to the development of new policies, introduction of new practices, and strengthening of current TB control policies and practices in Indonesia. However, the sustainability of these changes was often limited. The process of translating OR outcomes was complex. Even though OR findings are useful for TB control program improvement, the deployment of recommendations can be influenced by other factors. The skills and political power of researchers, interests and political power of policy makers, and health system response could all influence the process.

Therefore, we recommend the following. First, the OR initiative should be intensified and promoted. The curriculum of OR capacity building should include skills of knowledge translation and communication for advocacy. Second, the OR group should include health staff with sufficient power to achieve changes in the TB control program. Researchers with sufficient influence will contribute heavily to translating knowledge into policy. Third, the timing of advocacy actions should be carefully planned in order to increase the possibility of sustainable deployment of the recommendations. Fourth, short communication lines between the OR groups and policy makers or program managers during and after executing the OR projects ought to be established to ensure that the results and recommendations of the research are properly implemented. Finally, ‘OR on OR’ studies are required, particularly to explore potential mechanisms to sustain the impact of OR on policies and practices.
